# Whole-exome sequencing reveals Kawasaki disease susceptibility genes and their association with coronary artery lesion

**DOI:** 10.3389/fped.2024.1400123

**Published:** 2024-09-10

**Authors:** Yazhou Wang, Xuepeng Chen, Dufei Zhang, Renwei Chen, Ailixiati Alifu

**Affiliations:** ^1^ Department of Pediatric Vasculocardiology, Hainan Women and Children’s Medical Center, Haikou, China; ^2^ Department of Cardiothoracic Surgery, Hainan Women and Children’s Medical Center, Haikou, China

**Keywords:** Kawasaki disease, whole exome sequencing, coronary artery lesions, susceptibility genes, pediatrics - children

## Abstract

**Objective:**

This study aimed to explore Kawasaki disease (KD) susceptibility genes and their complications like coronary artery lesions (CAL) using whole exome sequencing (WES).

**Methods:**

Between April 1, 2021, and December 31, 2022, our study included 55 pediatric patients diagnosed KD at our center, alongside a cohort of healthy children who sought medical care at our institution during the same timeframe. We extracted peripheral blood DNA from all participants and employed the advanced high-throughput Illumina Next-Generation Sequencing technology for comprehensive analysis. Through bioinformatics evaluation, we identified potential susceptibility genes. Moreover, from the 55 KD patients, we selected 15 for the CAL group and 40 for the non-CAL group. We aimed to investigate whether there were significant differences in the allele frequencies of the targeted susceptibility genes between these subgroups, to explore the risk alleles associated with the development of CAL in KD.

**Results:**

HLA-DRB1 rs17882084 and IL6ST rs781455079 genotypes and alleles differed significantly between KD and non-KD (*P* < 0.05). No differences existed for IL17RC rs143781415 and VEGFB rs776229557 (*P* > 0.05). No differences in HLA-DRB1 rs17882084, IL6ST rs781455079, and VEGFB rs776229557 genotypes existed between CAL and non-CAL groups (*P* > 0.05). However, the IL17RC rs143781415 genotype differed significantly between them (*P* < 0.05).

**Conclusions:**

HLA-DRB1 rs17882084 and IL6ST rs781455079 genotypes may be potential KD susceptibility gene candidates. Specifically, HLA-DRB1 rs17882084 GA genotype and A allele, and IL6ST rs781455079 TC genotype and C allele may increase KD risk. Additionally, the IL17RC rs143781415 genotype may increase CAL risk in KD patients.

## Introduction

KD is an acute systemic vasculitis that primarily affects children, characterized by fever, rash, lymphadenopathy, edema of the hands and feet, oral mucosal changes, and non-suppurative conjunctivitis (1). While the exact cause of KD remains unclear, research suggests that genetic factors may play an important role in its pathogenesis (2). The most serious complication of KD is CAL, including coronary artery dilatation and aneurysms, which can lead to long-term cardiovascular issues (3).

With the development of genomics and bioinformatics technologies, WES has become a powerful tool for exploring susceptibility genes for various genetic diseases (4). By comparing the genomes of KD patients to those of healthy individuals, we can identify specific gene variants that may be associated with disease development.

This study aims to apply WES technology to analyze gene mutations in KD patients and compare them to gene data from healthy pediatrics. Through this approach, we hope to identify susceptibility genes associated with the pathogenesis of KD and its most severe complication, CAL.

## Materials and methods

### Study design and participants

Between April 1, 2021, to December 31, 2022, 63 children were continuously diagnosed with KD at Hainan Women and Children's Medical Center according to the 6th edition of the Diagnostic Guidelines for Kawasaki Disease (5). After rigorous screening, 8 children were excluded for not meeting inclusion criteria, leaving 55 children enrolled in the study. 55 healthy children matched by consultation date, gender, and age were selected from the Department of Child Healthcare at our Center during the same period as controls. KD children were divided into two groups based on the presence or absence of coronary artery lesions (CAL): CAL group and non-CAL group. The judgment criteria for CAL were based on the Canadian Dallaire database standards, with *Z* score ≥2.0 indicating abnormality (This definition includes temporary coronary dilation during the acute phase of KD and long-term coronary sequelae). Informed consent was obtained from the parents or guardians of all children, and the study was approved by the Medical Ethics Committee of Hainan Women and Children's Medical Center Inclusion criteria were: (1) children meeting the diagnostic criteria for typical KD; (2) informed consent signed by families; (3) complete medical records and ability to cooperate in completing the study. Exclusion criteria were: (1) children with infectious diseases, rheumatic diseases, or cardiovascular diseases; (2) children with a history of KD; (3) families declining participation; (4) children participating in other studies concurrently.

### DNA extraction, library preparation, and WES

Genomic DNA (gDNA) was extracted from the blood samples of the patients using the QIAamp DNeasy Blood &Tissue Kit (QIAGEN, Hilde, Germany). Genomic DNA of 1–3 μg was fragmented to an average size of 180 bp using a S220 Focused-ultrasonicator (Covaris, Massachusetts, USA), which was used to generate the index libraries (average size of 350–450 bp, with adapter) using the Library Preparation Kit (MyGenostics Inc.), according to the requirements of DNBSEQ platform. Target whole exome enrichment experiment was performed following the standard protocol of the GenCap Kits (MyGenostics Inc.). The enrichment libraries were sequenced on DNBSEQ-T7 sequencer for paired reading of 150 bp. The mean sequencing depth was over 100×.

### Bioinformatics analysis

We analyzed the shared genetic mutations and rare mutation genes in the WES data of 55 patients in the KD group. After sequencing, the raw data were saved as a FASTQ format. Both sequencing adapters and low quality reads (<80 bp) were filtered by cutadaptor software (http://code.google.com/p/cutadapt/). The clean reads were mapped to the UCSC hg19 human reference genome using BWA software (http://bio-bwa.sourceforge.net/). The duplicated reads were removed using Picard tools (http://broadinstitute.github.io/picard/), and the mapped reads were used for the detection of variation. The variants of SNP and InDel were detected by HaplotypeCaller of GATK software (https://software.broadinstitute.org/gatk/) and filtered by Variant Filtration of GATK software. Then, the data would be transformed to VCF format. Variants were further annotated by ANNOVAR software (http://annovar.openbioinformatics.org/en/latest/), and associated with multiple databases, such as, 1,000 genome (http://www.1000genomes.org/), ESP6500 (http://evs.gs.washington.edu/EVS), dbSNP (http://www.ncbi.nlm.nih.gov/projects/SNP/), EXAC (http://exac.broadinstitute.org/), Inhouse (MyGen ostics), HGMD (http://www.biobase-international.com/product/hgmd), and also predicted by SIFT (http://sift.jcvi.org/), PolyPhen-2 (http://genetics.bwh.harvard.edu/pph2/), MutationTaster (http://www.mutationtaster.org/), GERP++ (http://mendel.stanford.edu/SidowLab/downloads/gerp/index.html).Using the software CNVkit (https://github.com/etal/cnvkit)to perform CNV analysis on the sample, and detect the change of copy number based on the depth distribution of Reads compared to the reference genome. The pathogenicity classifcation of the sequence variation was performed according to the standards and guidelines for the interpretation of sequence variants recommended by the American College of Medical Genetics and Genomics and the Association for Molecular Pathology (6).

**Figure 1:**
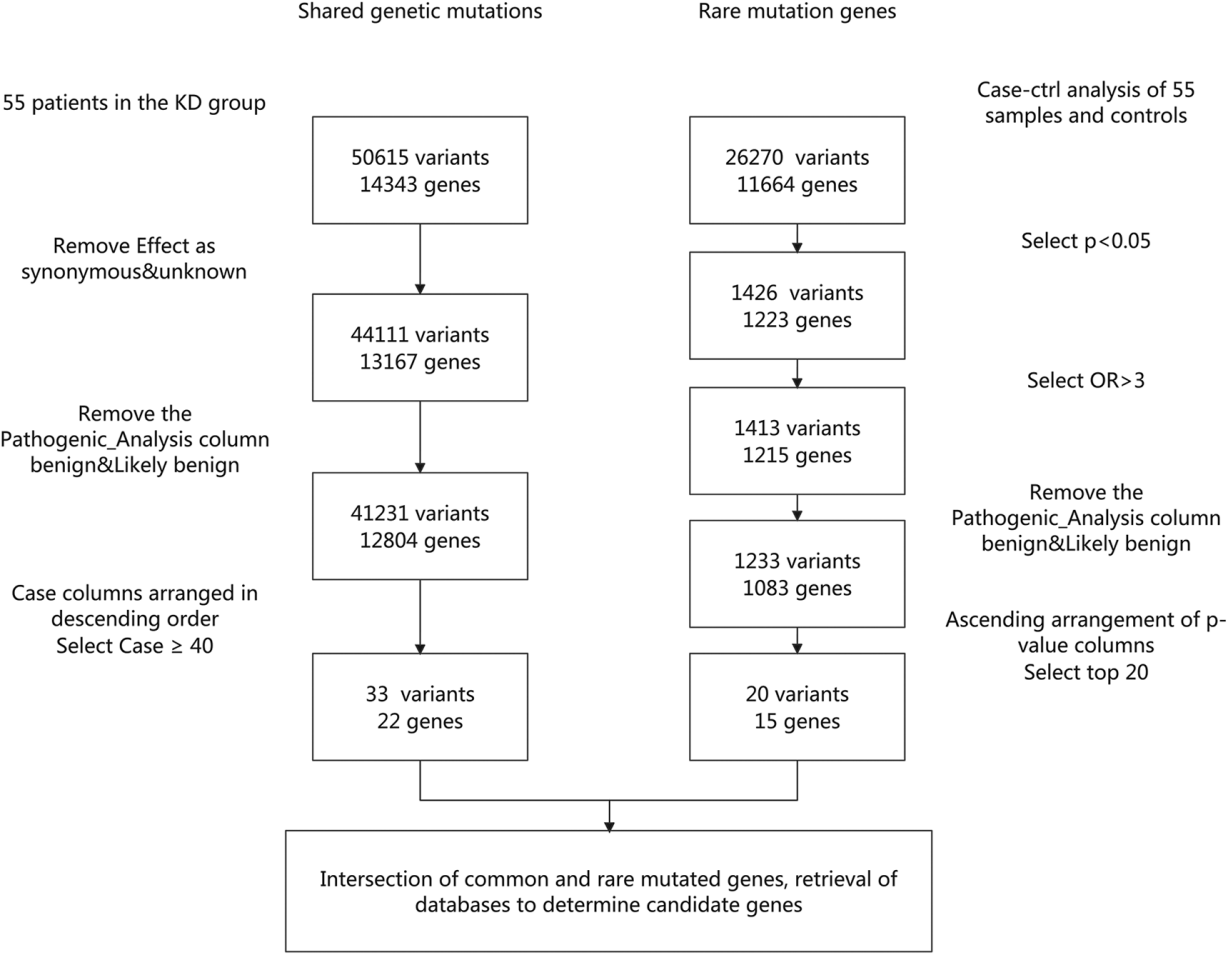
Workflow for variant filtration and selection of KD variants.

Based on the above analysis, among the 55 children with KD, 22 common genes with mutations (33 mutation sites) and 15 rare genes with mutations (20 mutation sites) were identified (Figure 1). We then intersected the 22 common genes with the 15 rare genes and identified the following genes: HLA-DRB1 rs17882084, IL6ST rs781455079, IL17RC rs143781415, VEGFB rs776229557, ITPKC rs76358638, CASP3 rs577739464, ORAI rs769061106, MYH11 (rs146388001, rs185661462, rs758895653, rs767136120), and SMAD9 (rs397514715, rs200651392). Although some mutation sites were detectable, their occurrence frequency was low (≤2 cases) and not statistically significant, so we selected mutation sites with an occurrence frequency of ≥3 cases. Ultimately, HLA-DRB1 rs17882084, IL6ST rs781455079, IL17RC rs143781415, and VEGFB rs776229557 were determined to be the candidate genes for this study.

### Statistical analysis

We utilized SPSS 18.0 statistical software for analysis. Categorical variables were displayed as proportions, while continuous variables were presented as mean ± standard deviation. The comparison of categorical variables between two groups was done using a χ^2^ test or Fisher's exact test. For numerical variables, Student's *t*-test or the Mann–Whitney *U*-test was used based on the distribution type. Effect size was reported as risk ratio or odds ratio with a 95% confidence interval. Statistical significance was defined as a *p*-value of <0.05.

## Result

The study included a total of 110 pediatric patients with an average age of 2.32 ± 1.51 years old, of which 48 were female. The KD group (*n* = 55) and the non-KD group (*n* = 55) had no significant differences in basic characteristics such as age, gender ratio, height, weight, etc. Among the KD group, 15 patients had coronary artery lesions and 40 patients had no coronary artery lesions (Table 1). There were no differences in age, gender, weight, and height between KD children with and without CAL lesions. Among the CAL group, the coronary artery lesion grades were as follows: 5 cases of grade II, 5 cases of grade IIIa, 3 cases of grade IIIb, and 2 cases of grade IV (Table 2).

**Table 1:** Baseline characteristics KD vs. non-KD.

Variables	KD (*n* = 55)	Non-KD (*n* = 55)	*P*-value
Age (years)	2.32 ± 1.52	2.33 ± 1.51	0.96
Gender (Male/Female)	31/24	31/24	NA
Height (cm)	86.64 ± 14.71	88.38 ± 15.23	0.28
Weight (kg)	11.80 ± 3.48	12.31 ± 3.55	0.34
Coronary artery lesion (cases)	15	–	–

**Table 2:** Baseline characteristics CAL vs. non-CAL.

Variables	CAL	Non-CAL	*P*-value
Age (years)	2.48 ± 1.80	2.26 ± 1.43	0.84
Gender (Male/Female)	10/5	21/19	0.35
Height (cm)	87.20 ± 17.96	86.43 ± 13.55	0.98
Weight (kg)	11.87 ± 4.25	11.77 ± 3.20	0.89
Coronary abnormalities grade	Grade II (*n* = 5), Grade IIIa (*n* = 5), Grade IIIb (*n* = 3), Grade IV (*n* = 2)	–	–

We analyzed the shared genetic mutations and rare mutation genes of 55 patients in the KD group. The specific screening process involved retrieving data from PubMed, DisGeNET, China National Knowledge Infrastructure and other databases to ultimately determine that HLA-DRB1 rs17882084, IL6ST rs781455079, IL17RC rs143781415, and VEGFB rs776229557 were candidate genes for this study (Figure 1).

The HLA-DRB1 rs17882084, IL6ST rs781455079 gene types and alleles showed differences in distribution between the KD group and non-KD group (*P* < 0.05). The HLA-DRB1 rs17882084 GA gene type and A allele, as well as the IL6ST rs781455079 TC gene type and C allele, were significantly more prevalent in the KD group compared to the non-KD group. The IL17RC rs143781415 and VEGFB rs776229557 gene types showed no differences between the KD group and non-KD (*P* > 0.05) (Table 3).

**Table 3:** Association of genotype/allele with kawasaki disease in KD and Non-KD groups.

SNP	Genotype/Allele	KD (*n* = 55)	non-KD (*n* = 55)	OR (95% CI)	*P*-value
HLA-DRB1 (rs17882084)	GG	39 (70.91%)	54 (98.18%)	16.00 (2.20–116.51)	0.0001
	GA	16 (29.09%)	1 (1.82%)		
	AA	0 (0)	0 (0)		
	G	94 (85.45%)	109 (99.09%)	16.00 (2.16–118.57)	0.0002
	A	16 (14.55%)	1 (0.91%)		
IL6ST (rs781455079)	TT	42 (76.36%)	55 (100%)	1.31 (1.13–1.52)	0.0001
	TC	13 (23.64%)	0 (0)		
	CC	0 (0)	0 (0)		
	T	87 (79.10%)	110 (100%)	1.13 (1.06–1.21)	0.0001
	C	13 (20.90%)	0 (0)		
IL17RC (rs143781415)	GG	51 (92.73%)	55 (100%)	0.93 (0.86–0.99)	0.13
	GA	4 (7.27%)	0 (0)		
	AA	0 (0)	0 (0)		
VEGFB (rs776229557)	GG	53 (96.36%)	55 (100%)	0.96 (0.92–1.01)	0.48
	GT	2 (3.64%)	0 (0)		
	TT	0 (0)	0 (0)		

SNP, single nucleotide polymorphism.

The distribution of HLA-DRB1 rs17882084, IL6ST rs781455079, and VEGFB rs776229557 genotypes were not significantly different (*P* > 0.05) between the CAL group and non-CAL group. The distribution of IL17RC rs143781415 genotype was significantly different (*P* < 0.05) between the two groups of KD patients (Table 4).

**Table 4:** Association of SNPs with CAL in KD.

SNP	CAL (*n* = 15)	Non-CAL (*n* = 40)	OR (95% CI)	*P*
HLA-DRB1 (rs17882084)	6 (40.00%)	10 (25.00%)	1.60 (0.71–3.63)	0.45
IL6ST (rs781455079)	2 (13.33%)	11 (27.50%)	0.49 (0.12–1.94)	0.46
IL17RC (rs143781415)	4 (26.67%)	0 (0)	1.36 (1.01–1.85)	0.005
VEGFB (rs776229557)	2 (13.33%)	0 (0)	1.15 (0.95–1.41)	0.07

SNP, single nucleotide polymorphism.

## Discussion

This study performed WES on KD patients and healthy children to explore genetic susceptibility genes associated with KD and its complication of CAL. Our findings revealed significant associations between HLA-DRB1 and IL6ST gene variants with KD disease risk, while IL17RC gene variation was related to the occurrence of CAL.

Onouchi Y et al. found a significant correlation between the ITPKC gene and the incidence of KD (7). In this study, we detected one case of the ITPKC (rs76358638) mutation in the KD group, consistent with their findings. Yoon KL analyzed 920 Japanese KD patients, 249 European-American KD patients, and healthy controls, discovering an association between the CASP3 gene and KD susceptibility (8). In our KD group, we identified two cases with the CASP3 (rs577739464) mutation, aligning with Yoon KL's findings. Onouchi et al. in a sibling-paired study, suggested a link between the CD40l gene SNP (rs1126535) and higher KD incidence in males (9). However, no mutations were found in this gene among the 31 male patients in our study, possibly due to our smaller sample size and genetic background differences, which require further investigation. Kariyazono H et al. found that the frequency of the VEGF g.-634 G>C single nucleotide polymorphism G allele in the promoter region was significantly higher in KD patients with CAL compared to those without CAL or healthy controls (10). In our study of 55 KD patients, we detected two cases with the VEGFB (rs776229557) mutation. Other studies have suggested that KD may be associated with mutations in the ORAI (rs769061106), MYH11 (rs146388001, rs185661462, rs758895653, rs767136120), and SMAD9 (rs397514715, rs200651392) genes. These loci were identified in our study as well, consistent with previous data. However, due to the small sample size, the number of cases detected was less than two, preventing effective statistical analysis.

Assari R et al. studied 55 Iranian KD patients and found the IL-6 (rs1800795) gene mutation in 27 cases, all with the GG genotype. In our study, 42 KD patients had the IL6ST (rs781455079) gene (11). Yang Y et al. analyzed 120 KD patients and 120 healthy children (control group) and found that the allele A at the rs3819025 locus of the IL-17A gene might be a risk factor for KD (12). Our study found that the IL17RC rs143781415 genotype might increase the risk of CAL in KD patients. Although the mutation sites differed, both IL-6 and IL-17, as pro-inflammatory factors, were highly detected in our study group, consistent with previous research. Tsai FJ et al. conducted a genome-wide association study (GWAS) in a Han Chinese population of 250 KD patients and 446 controls, and further validated in an independent cohort of 208 cases and 366 controls, identifying HLA as a novel KD risk gene (13). Our study found that the HLA-DRB1 rs17882084 genotype might be a susceptibility gene for KD, consistent with their findings. The GA genotype and A allele frequencies of the HLA-DRB1rs17882084 site were significantly higher in the KD group compared to controls. This suggests these genotype and allele may increase KD risk. HLAs exist mainly on cell membranes of B lymphocytes, monocytes, and endothelial cells (14). Multiple studies have identified HLA as a risk gene for KD, involved in T cell receptor-mediated signaling transduction, regulation of inflammatory cytokines, and antibody-mediated immune responses (13, 15). However, Huang et al. found that HLA-DRB1 alleles do not increase the occurrence of KD or CAL complications. Therefore, further research is needed to investigate the specific mechanisms by which HLA-DRB1 gene variations are involved in the pathogenesis of KD (16). However, currently, there is limited research on HLA as a biomarker related to Kawasaki disease (KD).

In KD, the immune system is activated, leading to an increase in cytokine production, which triggers an inflammatory cascade. The resulting large quantities of inflammatory factors can directly damage vascular endothelial cells. These damaged endothelial cells may highly express vascular endothelial growth factor (VEGF). VEGF can increase vascular permeability, promote endothelial cell proliferation, and superoxide production, leading to vascular damage and contributing to the development of coronary artery lesions (CAL) (17, 18). Suzuki et al. found that VEGF and its receptors were widely expressed in myocardial cells, vascular endothelial cells, and smooth muscle cells in children with Kawasaki disease (KD). This expression was particularly high in the thickened intimal smooth muscle of stenotic and recanalized vessels. In contrast, no VEGF expression was observed in the normal coronary arteries of individuals without a history of KD (19). No VEGF expression was observed in normal coronary arteries without a history of KD, indicating that VEGF expression is closely related to the formation of KD and vascular expansion. Autopsy results also revealed VEGF expression in coronary arteries. Immunohistochemical examinations and animal experiments confirmed that VEGF influences the occurrence of CAL (20, 21).

In this study, although the VEGFB rs776229557 genotype was detected in the KD group, there was no difference in the distribution of this genotype between the KD and control groups (*P* > 0.05). Additionally, the distribution of the VEGFB rs776229557 genotype showed no difference between KD children with and without CAL complications (*P* > 0.05). This suggests that the VEGFB rs776229557 genotype is neither a genetic factor for KD susceptibility nor a risk factor for the potentially fatal complication of CAL. Of course, this may be related to the relatively small sample size.

The TC genotype and C allele frequencies of the IL6ST rs781455079 site were significantly higher in the KD group than controls. This indicates this IL6ST gene variation may be another susceptibility factor for KD. IL-6 is a multifunctional cytokine synthesized by neutrophils and monocytes/macrophages, causing vasculitis injury (22). Many studies show IL-6 may be involved in KD pathogenesis and complicated CAL, with IL-6 potentially being an important factor causing immunological dysregulation in KD leading to CAL (23–25). However, our study suggests the IL6ST rs781455079 site variation did not increase risk of complicated CAL in KD. IL-6 is a pro-inflammatory factor involved in the proliferation, differentiation, survival of immune cells, and the production of inflammatory mediators. Studies have found that IL-6 can serve as a potential biomarker for the early diagnosis of Kawasaki disease (KD). IL-6 promotes the expression of adhesion molecules in endothelial cells, stimulates platelet activation, and mediates the proliferation of vascular smooth muscle cells, directly contributing to the development of coronary artery lesions (CAL). The severity of CAL is positively correlated with IL-6 levels. Therefore, IL-6 not only holds potential value in the early diagnosis of KD but also may become an important indicator for predicting the risk of CAL (23, 26).

Notably, we also found a significant difference in IL17RC rs143781415 genotype distribution between KD patients with and without CAL. This suggests this genotype may increase the risk of CAL in KD patients. IL-17 is produced by various immune cells including CD4+ T helper cells, especially the Th17 subset. Th17 cell differentiation and activation is regulated by many factors. Once activated, Th17 cells produce inflammatory factors like IL-17, participating in regulation of inflammatory reactions (15). Overexpressed IL-17A binds its receptor IL-17RA, exerting strong pro-inflammatory effects via NF-kB and MAPK pathways, involved in complicated CAL in KD (27). IL-17 is a pro-inflammatory factor produced by various immune cells, including subsets of T cells, particularly the CD4+ T helper cell subtype known as Th17 cells. The differentiation and activation of Th17 cells are regulated by multiple factors, and once activated, they can produce IL-17 and other inflammatory mediators, participating in the regulation of inflammatory responses. Research has found that in the early stages of Kawasaki disease (KD), excessive activation of the immune system leads to an increase in Th17 cells and the release of IL-17, triggering an inflammatory response. IL-17 activates various target cells, such as endothelial cells and fibroblasts, inducing the production of inflammatory mediators and causing inflammatory damage to the vascular walls. Therefore, IL-17 may become a new biomarker for the early diagnosis of coronary artery damage in KD (28).

However, our study has some limitations. Firstly, the relatively small sample size may limit statistical power to uncover more potential susceptibility genes. Secondly, while we identified specific gene variations associated with KD and CAL, KD pathogenesis involves interactions between multiple genetic and environmental factors. Future research requires larger sample sizes and multicenter collaboration to further validate these findings and explore how these gene variations interact with other genetic and environmental factors to cause KD and its complications.

## Conclusion

Our study provides new insights into the genetic basis of KD and its complication CAL, as well as valuable information for future research into pathological mechanisms and potential therapeutic targets. However, more research is needed to further elucidate the specific relationships between these gene variations and KD pathogenesis, and how they impact disease progression and prognosis.

## Data Availability

The data reported in this paper have been deposited in the OMIX, China National Center for Bioinformation/Beijing Institute of Genomics, Chinese Academy of Sciences (https://ngdc.cncb.ac.cn/omix: accession no. OMIX007257).
